# State-of-the-Art Sensor Technology in Spain: Invasive and Non-Invasive Techniques for Monitoring Respiratory Variables

**DOI:** 10.3390/s100504655

**Published:** 2010-05-05

**Authors:** Christian Domingo, Lluis Blanch, Gaston Murias, Manel Luján

**Affiliations:** 1 Pneumology Service, Hospital de Sabadell, Corporació Parc Taulí, 08208 Sabadell, Spain; 2 Department of Medicine, Autonomous University of Barcelona (UAB), 083208 Bellaterra, Barcelona, Spain; 3 Critical Care Center; Hospital de Sabadell, Corporació Parc Taulí, 08208 Sabadell, Spain; E-Mail: lblanch@tauli.cat; 4 Institut Universitari Fundació Parc Taulí, Corporació Parc Taulí Autonomous University of Barcelona (UAB). 08208 Sabadell, Spain; 5 Intensive Care Unit, Clínica Bazterrica and Clínica Santa Isabel. Buenos Aires, Argentina; E-Mail: gaston@gmail.com; 6 CIBER Enfermedades Respiratorias CIBERes, Spain

**Keywords:** physiological parameters, transcutaneous arterial oxygen saturation, transcutaneous partial pressure of carbon dioxide, capnometry, mechanical respiratory parameters, mechanical ventilation, home care, telemonitoring

## Abstract

The interest in measuring physiological parameters (especially arterial blood gases) has grown progressively in parallel to the development of new technologies. Physiological parameters were first measured invasively and at discrete time points; however, it was clearly desirable to measure them continuously and non-invasively. The development of intensive care units promoted the use of ventilators via oral intubation ventilators via oral intubation and mechanical respiratory variables were progressively studied. Later, the knowledge gained in the hospital was applied to out-of-hospital management. In the present paper we review the invasive and non-invasive techniques for monitoring respiratory variables.

## 1. Introduction

In the 1950s, reports of blindness caused by oxygen therapy administered to premature babies drew attention to the need to develop non-invasive techniques for measuring PaO_2_. Later, clinicians became interested in measuring PaCO_2_ as well. An important milestone was the introduction of pulse oximetry in 1985, which provided effective non-invasive assessment of oxygenation during sleep and exercise. Pulse oximetry cannot, however, provide information on PaCO_2_, so it cannot provide information about the patient’s ventilation. Therefore, the development of techniques for non-invasive monitoring of PaCO_2_ became a priority.

Interest in measuring physiological parameters grew progressively in parallel with the development of new technologies and intensive care units (ICU). Physiological parameters were first measured invasively and at discrete time points; however, it was clearly desirable to measure these parameters continuously and non-invasively. The development of ICUs promoted the use of ventilators via oral intubation, and mechanical respiratory variables were progressively studied. Later, the knowledge gained in the hospital was applied to out-of-hospital management, and devices were developed designed to provide ambulatory care for chronic diseases [1–3], further increasing the interest in non-invasive monitoring methods. In the last two decades, telemedicine has developed in parallel with new technology (sensors and devices) [4,5]. Telemonitoring is emerging as a highly promising way of managing the large numbers of patients with chronic illness [6,7]. The most sophisticated telemedicine systems offer real-time visualization of patients at home and of their physiological data [8,9].

## 2. Non-Invasive Measurement of Blood Gases

In the pulmonary field, arterial blood gases and airflow are the target parameters to be measured. Techniques for assessing arterial blood gases were first developed for measuring oxygenation [10,11] and then for measuring PaCO_2_ as well [12,13]. However, interest in non-invasive measurement of both PaCO_2_ and PaO_2_ gradually faded in view of the technical and practical problems associated with the measuring devices used (Table 1) [11].

### 2.1. Arterial Oxygen Measurement

The physiological basis of blood gas pressure determination through the arterialization of capillary blood derives from experiments conducted by Baumberger and Goodfriend more than 50 years ago [14]. These authors demonstrated that if the surface of human skin is heated to the maximum supportable temperature (approximately 45 °C), the pressure of oxygen in the skin increases to approach that of the pressure in arterial blood. Twenty years later, Huch *et al*. showed that PaO_2_ in premature and newborn babies could be measured with an electrode heated to 43 °C to 44 °C [15]. Interest was initially focused on determining PaO_2_ transcutaneously, as this was the only non-invasive alternative to invasive arterial gasometry. However, the development of pulse oximetry largely supplanted the traditional transcutaneous determination. Moreover, recent studies have shown that transcutaneous measurement systematically underestimates arterial PaO_2_ [16]. Despite the controversy surrounding the accuracy of transcutaneous oxygen measurement, in certain countries such as Germany, Switzerland and Austria transcutaneous oximetry based on the arterialization of capillary blood is widely used in neonatal ICUs [17].

Although the gold standard continues to be invasive blood gas monitoring through arterial puncture, pulse oximetry has proven effective for non-invasive assessment of oxygenation during sleep [10] as well as during exercise [11], and has become widely used since its introduction in 1985. Several factors, including race, smoking, and jaundice may influence pulse oximeter readings [11]. Recently, during the validation of a new pulse oximeter [12], we observed that oxygen saturation readings became reliable only two minutes after placing the device, although in clinical practice readings are often recorded almost immediately. Pulse oximetry cannot, however, measure PaCO_2_, so it cannot inform us about the patient’s ventilation.

### 2.2. Arterial CO_2_ Measurement

Whereas non-invasive arterial oxygen saturation monitoring using pulse oximetry has proven reliable, non-invasive monitoring of PaCO_2_ remains controversial. Two techniques are of special interest in the non-invasive estimation of PaCO_2_: capnography (based on capnometry) and transcutaneous CO_2_ measurement.

#### 2.2.1. Capnometry-Capnography

Capnography has long been the standard of care for the non-invasive estimation of PaCO_2_ in many health disciplines, including anesthesiology, and pulmonary, critical care, and emergency medicine. Capnometry refers to the continuous measurement of CO_2_ in exhaled air, which is often accomplished by infrared spectrometric analysis during the respiratory cycle. All capnographs provide a single value for CO_2_, usually from end-respiration to end-respiration, designated end-tidal CO_2_ (EtCO_2_). When capnometric values are displayed as breath-to-breath CO_2_ elimination curves, the technique is referred to as *capnography*.

Two main types of capnographs are commonly available. The first, *mainstream capnometry*, uses a sensor placed directly in the airway through which the patient exhales – in most cases, the orotracheal tube. The signal generated is analyzed in the sensor itself and then electronically transmitted to the device, which shows the readings on a screen. The second type, *sidestream capnometry*, continually aspirates samples of expired air to be processed in the device [13]. Although no significant differences between measurements from the two types have been found*,* mainstream capnometry is more common in critical patients because its response is quicker and it has none of the complications derived from the aspiration of air (mainly due to obstruction of the device).

##### 2.2.1.1. Physiological Basis for Capnography Interpretation

Despite the relatively simple technology behind capnography and the ease with which capnographic measurements are obtained, the interpretation of the results requires a profound knowledge of the physiological bases of CO_2_ production, transport, and elimination.

*CO_2_ production*. CO_2_ is the end product of aerobic metabolism in tissues. In normal conditions, the ratio of CO_2_ production to oxygen consumption (the respiratory coefficient) is 0.8. However, tissue CO_2_ production increases in certain situations (fever, intense physical exercise, convulsions, or hyperthyroidism, among others) and decreases in others (sedation, hypothermia, and hypothyroidism).

*Transport*. CO_2_ is transported in three fundamental ways: directly dissolved in blood (making up 10% of venous blood), as a bicarbonate anion (75%–80%), and as carbamino compounds (10%). Fluctuations in cardiac output will thus influence CO_2_ transport until it is eliminated through ventilation. Thus, CO_2_ accumulates in the venous compartment when cardiac output is low and CO_2_ accumulation is greatest during circulatory arrest.

*Elimination*: CO_2_ is eliminated through the lungs by alveolar ventilation. Therefore, appropriate CO2 elimination requires the integrity of the components involved in ventilation and perfusion. The components involved in ventilation can be divided into the portion of the airway that intervenes in gas exchange (alveolar) and the portion of the airway that does not intervene in gas exchange (dead space). The dead space comprises the conducting airways of the respiratory tract (anatomic dead space) and areas that are ventilated but that do not participate in perfusion (physiological dead space).

Thus, the value of EtCO_2_ depends on extrapulmonary factors (endogenous tissue production and transport of CO_2_ to the lungs) as well as pulmonary factors (CO_2_ clearing by alveolar ventilation). In baseline conditions (cardiac output within the normal ranges, absence of situations causing hyperproduction or underproduction of cellular CO_2_), the gradient between PaCO_2_ and EtCO_2_ − P(a-et)CO_2_, see below–ranges between 1 to 3 mmHg. Changes in the P(a-et)CO_2_ gradient have been studied in several clinical and experimental situations. In an animal model of hemorrhagic shock [18], progressive exsanguination decreased cardiac output and the diminished capacity of transport of CO_2_ to the lungs was reflected in lower than expected EtCO_2_ values. The P(a-et)CO_2_ gradient peaked in advanced phases of exsanguination (up to 10 mmHg).

##### 2.2.1.2. The capnogram: the Graphical Representation of Capnometry, the Normal Capnogram

**Figure 1.**
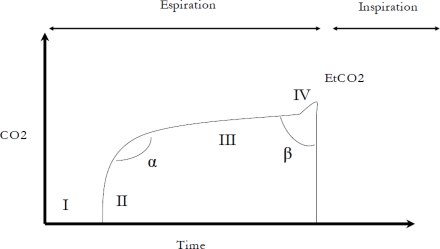
Phases and angles of a capnogram.

Capnograms can show ventilation in one of two ways: graphs resulting from plotting CO_2_/time and those resulting from plotting CO_2_/volume. The usual representation is CO_2_/time, with PaCO_2_ on the X-axis and time on the Y-axis. *Phases of the capnogram*: the shape of the capnogram usually has three (optionally four) phases and two angles (Figure 1).

Phase I represents the clearing of the dead space at the beginning of an expiration; in normal conditions, the dead space is void of CO_2_. In phase II, the value of CO_2_ increases abruptly, reflecting the transition from clearing the large caliber airways to clearing the first alveolar units. Phase III, the alveolar plateau, which lasts throughout most of the expiration, reflects the CO_2_ content of millions of alveolar units each with different CO_2_ concentrations. Thus, both phase III and the value of CO_2_ at the end of phase IIl (EtCO_2_) are products of the CO_2_ mixture contained in these millions of alveolar units. Occasionally, a fourth phase (phase IV) in which an abrupt increase in CO_2_ is seen at the end of phase III is encountered in patients with conditions like pregnancy or significant obesity [19].

*Angles in the capnogram*. In normal conditions, the angle formed between phases II and III (alpha) is approximately 110°. This angle can increase in certain situations; for example, the slope of phase III of the capnogram typically increases in patients with altered ventilation-perfusion (increased phase III slope basically indicates a lack of homogeneity). The second angle in the capnogram (beta) occurs between phase III and the start of the inspiration (CO_2_ = 0). In normal conditions, the beta angle is about 90 to 110 degrees; however, it can increase in situations where CO_2_ is reinhaled (for example, when non-invasive ventilation is supplied through a single tube without an expiratory valve). In addition to being able to recognize the normal shape of the capnogram, physicians need to be familiar with the most common abnormal shapes that can occur. Table 2 summarizes the most common capnographic abnormalities and the situations in which they usually arise.

##### 2.2.1.3. Volumetric Capnography. The Importance of the P(a-et)CO_2_ Gradient

**Figure 2.**
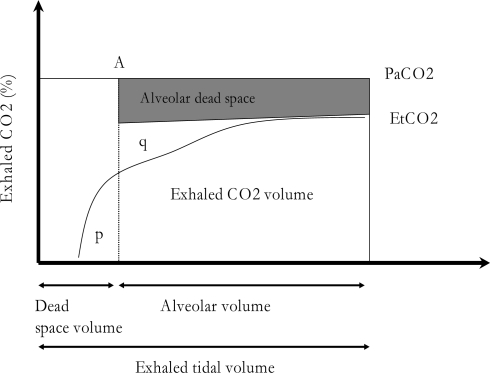
The measurement of exhaled and arterial CO_2_ makes it possible to represent the dead space.

An alternative way of graphically representing capnometric information is by plotting expiratory CO_2_ on the X-axis and volume on the Y-axis. These capnograms are obtained by incorporating a pneumotachograph to the capnograph in order to provide continuous measures of CO_2_ elimination as a function of the volume of gas expired. The combination of airflow and capnography monitoring allows calculation of breath by breath CO_2_ production and pulmonary dead space. Therefore, the use of volumetric capnography is clinically more useful than time-based capnography. As shown in Figure 2, there is a procedure for measuring physiological dead space based on the geometric method of equivalent areas (p = q), obtained by crossing the back extrapolation of phase III of the expired CO_2_ concentration over time with a vertical line traced so as to make the p and q areas equal. Airway dead space is then measured from the beginning of expiration to the point where the vertical line crosses the volume axis.

Estimating the dead space is important for determining ventilator settings in patients with ARDS. Positive end-expiratory pressure (PEEP) is used to increase lung volume and to improve oxygenation in these patients. However, this technique may be helpful or harmful. On the one hand, PEEP recruits collapsed lung units and improves oxygenation, thus reducing the alveolar dead space since alveolar recruitment is associated with decreased P(a-et)CO_2_; on the other, PEEP-induced overdistension may increase dead space and widen this difference. Thus, the P(a-et)CO_2_ gradient reflects the physiological dead space. In ideal ventilation-perfusion conditions, the value of EtCO_2_ is situated between 1 and 3 mmHg lower than the PaCO_2_ measured in arterial blood; this difference is known as the P(a-et)CO_2_ gradient. However, in some situations this gradient can suffer considerable variations, and it is crucial to take these situations into account when monitoring critical patients by capnography. Table 2 shows the main causes of increases in the P(a-et)CO_2_ gradient (PaCO_2_ more than 3 mmHg higher than EtCO_2_) and of decreases in the gradient (PaCO_2_ less than 1 mmHg higher than EtCO_2_; the gradient can even be negative (EtCO_2_ > PaCO_2_)).

Generally speaking, the P(a-et)CO_2_ gradient increases in conditions that increase dead space; in other words, P(a-et)CO_2_ is proportional to the dead space/tidal volume coefficient (VD/VT). Thus, diseases like chronic obstructive pulmonary disease or even conditions characterized by low cardiac output [20] cause an increase in ventilation respect to perfusion and an increased P(a-et)CO_2_. On the other hand, negative gradients can occur in situations involving hyperproduction of CO_2_ (fever, sepsis, hyperthyroidism, exercise) and even in expiration maneuvers prolonged beyond the tidal volume, especially in spontaneously ventilated patients (see below). Both possibilities must be kept in mind, both at the start of monitoring and when interpreting changes in either direction. For example, a drop in EtCO_2_ in a patient without significant changes in ventilation can indicate low cardiac output, whether due to hypovolemia or to heart failure from other causes. On the other hand, in a patient with fever and a gradient of 3 mmHg, EtCO_2_ may increase to values greater than PaCO_2_, resulting in a negative gradient. Likewise, it is important to bear in mind that EtCO_2_ reflects the “peak” concentration of CO_2_ in a few particular alveolar units, mainly with a low V/Q ratio (but greater than 0, given that they are reflected in the capnogram), whereas PaCO_2_ is the mean product of millions of alveolar units with different V/Q ratios. Thus, the P(a-et)CO_2_ gradient is essentially an indicator of alterations in ventilation/perfusion with mainly cardiopulmonary causes and is directly proportional to the degree of physiological dead space.

In spontaneously breathing patients, several attempts have been made to study the correlation between PaCO_2_ and EtCO_2_ in different clinical settings such as pulmonary embolism [21] and in patients with a variety of pulmonary diseases in an emergency department [22]. Moreover, in spontaneously breathing patients, the breathing pattern and especially the depth of the expiratory maneuvers have also been found to influence the P(a-et)CO_2_ gradient [23].

There may be alveolar units with a prolonged time constant due to bronchial narrowing which do not empty during tidal breathing. The CO_2_ of these alveolar units tends to equilibrate with mixed venous P CO_2_ values. In a longer expiratory maneuver, the greater expiratory time may allow lung units with low ventilation-perfusion ratio (V/Q) to empty and to be represented in a capnogram. This phenomenon has also been described by other authors [13–30].

#### 2.2.2. Transcutaneous CO_2_ Measurement

The basis for the transcutaneous measurement of PaCO_2_ was first described by Severinghaus *et al*. in the 1960s. These authors demonstrated that there is a linear relationship between cutaneous and true PaCO_2_ in the range of 20 to 75 mmHg. At the turn of the new century, Rohling and Biro [24] and Tschupp and Fanconi [25] published the first articles on monitors that incorporated the elements of an optical pulse oximetry sensor with a Severinghaus-type PaCO_2_ sensor [26]. Today, the transcutaneous non-invasive estimation of arterial PaCO_2_ is commonly accepted and widely used.

The main technical problems in the systematic use of transcutaneous PaCO_2_ are due to the electrochemical nature of the commercially available sensors. Briefly, there are two main problems: the learning process (the proper placement of the sensor and the management of the device require a minimal degree of expertise) and the consumption of materials such as membranes or gas for calibration. These sensors need systematic calibration before each measurement and the membrane of the electrochemical sensor needs to be changed after a period of time that varies between the different devices; while some companies indicate that membranes should be changed before each measurement, others recommend that they should be changed every two weeks).

**Figure 3.**
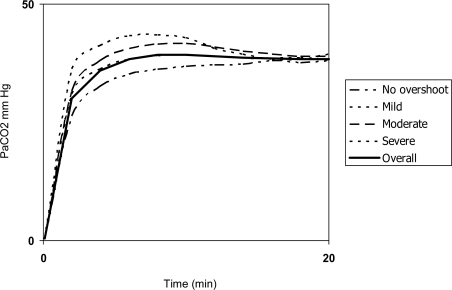
Graphic representation of the overshoot phenomenon.

Measurements of transcutaneous CO_2_ can be used in two ways, each of which has some particular features of its own:

a) As a non-invasive substitute for arterial blood gases for discrete measurements in the pulmonary function laboratory or in emergency departments. In this setting, a key point that remains to be established is the point in time when the monitor offers the most reliable measurement, which can be influenced by the “overshoot phenomenon”: two opposing phenomena may interfere with TcCO_2_ readings during the first few minutes of monitoring. First, the arterialization of capillary blood due to vasodilation takes several minutes. Second, applying heat increases the metabolism of the cells in the surface of the skin, raising CO_2_ release from these cells and causing a transitory overestimation of true PaCO_2_ (Figure 3). Overshoot usually precedes the complete vasodilation of subdermal tissue [27].

Overshoot seems to vary among individuals and therefore cannot be predicted before monitoring. Early high TcCO_2_ readings may warn the clinician of its presence, although their interpretation depends on the patient’s baseline PaCO_2_ [28].

b) As a monitoring tool that provides continuous estimates of arterial PaCO_2_. In this setting, two main drawbacks should be mentioned:

The delay in detecting acute events, which is estimated at about 2 min. This means that short events, like sleep apneas, often go undetected by the device.

Shifts during prolonged periods of monitoring. In some devices, the magnitude of the shift may reach values as high as 1.3 mmHg/hour of monitoring. Some commercial devices systematically correct the shift, but it is important that staff that manage the device are aware of it.

Despite these limitations, transcutaneous CO_2_ monitoring may be a useful adjunct in various clinical scenarios and should help improve control and therapy in many patients in the coming years [29]. Optical sensors may overcome some of the drawbacks associated with the current electrochemical sensors; they determine CO_2_ by measuring its optical absorption in the evanescent wave of a waveguide integrated in their surface. In a preliminary study [30], the accuracy of optical sensors was similar to that of commercially available electrochemical sensors. Optical sensors have the following advantages over electrochemical sensors: they do not require systematic calibration and membrane replacement; there is no shift, and their response is faster. Therefore, it is likely that optical sensors will supplant electrochemical sensors in transcutaneous monitoring of arterial blood gases.

### 2.3. Combined Estimation of PaO_2_ and Arterial CO_2_

Certain commercially available monitors offer transcutaneous measurement of both transcutaneous CO_2_ and O_2_. Many of these devices also allow the physician to set the working temperature of the electrode applied to the skin. Thus, while transcutaneous pO_2_ measurement can require working temperatures of 45 °C, temperatures between 41 °C and 42 °C suffice for TcCO_2_ measurement, although some authors have reported that TcCO_2_ measurements are more accurate at higher temperatures and recommend 43 °C for measuring both transcutaneous CO_2_ and O_2_. Nevertheless, as stated above, even at high temperatures, the correlation between transcutaneous O_2_ and PaO_2_ is suboptimal.

In a previous study our group validated a combined ear-lobe sensor SpO_2_/ transcutaneous CO_2_ [3], finding a good correlation between TcCO_2_/PaCO_2_ and SpO_2_/SaO_2_. We also determined that the monitor needed a maximum stabilization time of 17 minutes to allow PaCO_2_ readings. In later work we established that the ideal time to obtain readings ranged from 10 to 12 minutes, due to the overshoot phenomenon.

## 3. Physiologic Parameters Evaluated during Mechanical Ventilation

In mechanically ventilated patients, respiratory mechanics can be measured in dynamic (no flow interruption) or static (occlusion technique) conditions. Modern ventilators employed in ICUs measure breath-by-breath flow (*V̇*), volume (V), and pressure at the airway opening (Pao) and display the information graphically in real time as curves against time or as combined loops. Data obtained from curves analysis can help physicians understand the complex interactions between patients and ventilators.

Assisted ventilation can be total, partial, or absent, depending on the respiratory muscles’ ability to generate the pressure applied to the respiratory system (Prs). The Prs of a ventilated patient is the sum of the pressure generated by the ventilator Pao and the pressure developed by the respiratory muscles (Pmus). The latter is negative, as inspiratory muscles act by producing a decrease in pressure below the airways, as described by the equation of motion [31–33].

$$\text{Pr}\;s=\mathrm{Pao}+(-\mathrm{Pmus})=\dot{V}\times R+\frac{V}{C}+k$$

where V is the volume, *V̇* the flow over time, and k is a constant representing the alveolar end-expiratory pressure. The term *V̇* × *R* corresponds to the pressure dissipated across the airway and the endotracheal tube to overcome the frictional forces generated with gas flow (Pres). The rate of Pres and *V̇* defines the resistance of the respiratory system (Rrs).

The term $\frac{V}{C}$, on the other hand, corresponds to the pressure that must be applied to overcome elastic forces (Pel), which depends on both the volume insufflated in excess of resting volume (Vr) and on the respiratory system compliance (Crs). The constant k takes into account the application of positive end-expiratory pressure (PEEP) or intrinsic PEEP (PEEPi), if present. When the patient’s breathing activity is entirely passive, Pmus is negligible, and the driving pressure necessary to move air in and out of the thorax can be described by the simplified equation of motion [31–33]:

$$\text{Pr}\;s=\mathrm{Pao}=\dot{V}\times R+\frac{V}{C}+k$$

### 3.1. Static Mechanics

During mechanical ventilation the rapid airway occlusion method is the most commonly used technique for measuring respiratory mechanics. The mean airway pressure (Paw) wave has a characteristic trend with the highest peak at end-inspiration (Pmax), followed by a rapid drop after the occlusion (P1), and a slow decline until a plateau is reached (P2). P2 is the static pressure of the respiratory system (Pst, rs), which, in the absence of flow, equals the alveolar pressure (Palv), reflecting the elastic retraction of the entire respiratory system. The pressure drop from Pmax to P1 represents the pressure dissipated by the flow-dependent resistances. The slow decline from P1 to P2 after the occlusion depends on the viscoelastic properties of the system and on the pendulum-like movement of the air. In normal subjects, the alveolar pressure is near zero at end-expiration. Limiting expiratory flow or an inadequate respiratory pattern (high tidal volume or high respiratory rate) causes PEEPi due to volume trapping. PEEPi is detectable during the post-expiratory occlusion maneuver. The rapid airway occlusion technique is quick and easy to perform and provides immediate clinical data; this is especially useful when monitoring a paralyzed patient, even with a nonconstant flow wave, as occurs during pressure-controlled ventilation. Plateau pressure (P2) can be measured by applying a pause at end-inspiration during pressure-controlled ventilation [34–37].

#### 3.1.1. Time-Course of Paw during Constant Flow Inflation (Volume-Controlled Ventilation)

Observing the pressure wave in the airway as a function of time during constant-flow ventilation in the absence of spontaneous inspiration provides information about the elastic load (tension) to which the lung parenchyma is subjected. During inspiration with constant airflow, there is a linear increase in flow volume. Therefore, during the constant-flow inspiratory phase, changes in the concavity or convexity of the pressure wave are associated with unnecessary stretching/overdistension of ventilated alveolar regions or to recruitment and derecruitment of alveolar regions that were collapsed at the start of inspiration and opened during inspiration. A linear pressure wave during inspiration indicates constant compliance and the absence of overdistension and/or cyclic recruitment and derecruitment of alveolar regions [33,35,38]. The mathematical analysis of the shape of the dynamic pressure wave in the airway during constant-flow ventilation has led to the coining of the term “stress index”, which can be analyzed breath to breath. After physiological studies in animals showed that a nonlinear pressure wave in the airway was associated with ventilator-induced lung injury [38], studies in ALI/ARDS patients confirmed these preliminary experimental data. Several authors have found that a sustained pattern of overdistension is associated with greater pulmonary inflammation and worse prognosis [39,40]. Currently, no computerized system is available for continuous monitoring of the stress index. No automatic methods have been developed to analyze the data stored and this information cannot be integrated into the electronic clinical history.

### 3.2. Dynamic Mechanics

Dynamic mechanics may be derived during spontaneous breathing and in intubated patients in partially or totally supported ventilation [41]. In patients ventilated with partial support, the real-time analysis of the curves displayed can provide a great deal of useful information.

Patient-ventilator synchrony is achieved using a flow- or pressure-trigger system. In pressure-trigger mode, the ventilatory support detects the drop in airway pressure that occurs during inspiratory efforts that surpass a specific threshold value (usually 0.5–1 cmH_2_O during assisted ventilation, although higher threshold values of up to −20 cmH_2_O may be set). Inappropriate trigger sensitivity may lead to increased work of breathing, delaying weaning, or patient-ventilator dyssynchrony, and subsequent need for sedation. On the other hand, flow triggering has the advantages of lower inspiratory workload and greater sensitivity, but it sometimes triggers ventilatory support when patients are not spontaneously breathing (autotriggering) [42,43], which may also result from factors such as leaks in the circuit, expiratory fluctuations caused by water in the circuit, and cardiogenic oscillations [44].

A correct understanding of flow-trigger ventilation is important when managing non-invasively ventilated patients. Using masks or helmets during non-invasive ventilation (NIV) causes frequent air leaks and patient ventilator asynchronies during the inspiratory phase. This problem can be overcome by setting the bias flow as a compensating system, since its capacity reaches 20 liters/min; thus, PEEP can be applied without causing autotriggering [37,45].

Ineffective inspiratory efforts are the most common manifestation of poor patient-ventilator interaction [45]. The contraction of the inspiratory muscles against a closed valve has a high metabolic cost and can lead to the expression of inflammatory mediators in the muscles of the diaphragm [46,47]. Detecting ineffective inspiratory efforts should be a priority during mechanical ventilation, and ventilator settings (mode, airflow, tidal volume, expiratory time, and PEEP) should be adjusted to minimize them. A significant number of ineffective efforts is associated with increased need for sedation, episodes of arterial desaturation, behavioral disorders (delirium, episodes of panic, *etc.*), longer mechanical ventilation, and longer ICU stays [48,49].

The number of inspiratory efforts that a patient makes in a specific period of time can only be quantified by continuous observation of the airflow and airway pressure waves on the respirator’s screen or with specific algorithms [50,51].

#### 3.2.1. Signal Acquisition from Medical Devices

Up to 77% of admissions to medical ICUs take place, at least in part, for the purposes of monitoring, although only 10% of the patients monitored will subsequently have indications for major interventions [52,53]. Modern ICU equipment takes advantage of a wide range of technologies to track physiological variables in order to detect potentially life-threatening changes. As response time is a key issue, most of these devices are equipped with a more or less sophisticated set of alarms that alert intensivists, pulmonologists, nurses, or respiratory therapists about changes that could represent a risk to patients [54].

Current monitoring systems show waves and values. Even when alarm thresholds are based on values, analyzing waveforms can be very useful. Specialists tend to analyze waveforms at the bedside. For example, in the intracranial trace, a P2 wave greater than the P1 wave can alert the specialist to a drop in intracranial compliance. Persistent airway flow at end-expiration is synonymous with autoPEEP. Extreme variability in pulse pressure suggests the need for intravenous fluid replacement, and so on. However, physicians require extensive training in order to carry out these analyses. It may be relatively simple to implement automatic processes for evaluating these phenomena once the signals from the monitoring devices have been acquired by a centralized system. To achieve this, information must be available in a form that is independent of the manufacturer of the monitor or ventilator.

The combination of elements from different monitoring devices (pressure at airway opening, airflow, and capnograms) could be of clinical interest. Measuring the volume of expired CO_2_ (rather than the pressure of CO_2_ in expired gases, which is usually available) allows the approximate state of the patient’s metabolism to be estimated, and some parameters calculated from volumetric capnography provide information about the condition of the lungs and cardiovascular function. If the information from the ventilator flow sensor were combined with information from the patient’s capnograph (nearly always available in mechanically ventilated patients), information about the volume of expired CO_2_ would be easily available without the need for additional equipment [55–57].

Because manufacturers of monitoring systems are concerned about avoiding false negatives (a true event going undetected), they lower the alarm thresholds, thereby generating excessive, often useless, information. Kestin et al. showed that, in the operating room, where patients are normally sedated and relaxed, only 3% of the alarms that went off alerted to events that represented a true risk to the patient [54]. In the ICU, where the manipulation of patients often causes false detections, the situation is probably worse, leading to a high rate of false alarms and high level of noise [58,59].

Incorporating monitoring systems with more complex algorithms might represent a considerable improvement. Currently, signals are treated as if they were completely unrelated. Even when the ECG and respiratory plethysmographic signals are measured by the same sensors, the malfunction of one of the electrodes usually triggers a low level ECG alarm (“Electrode disconnected”) and a maximum level alarm in the plethysmograph (“Apnea”). A more comprehensive overview is necessary to understand the physiological and physiopathological phenomena involved. For example, the combined presentation of bradycardia, hypertension, and increased intracranial pressure means much more than the sum of each individual phenomenon. While each element might result from many causes, the level of uncertainty is much lower when the three alterations present together.

The final aim of alarm programming is patient safety. In an environment in which human resources are scarce, simply warning of a potentially dangerous event does not guarantee that this aim will be fulfilled. Therefore, incorporating expert knowledge in monitoring systems could help staff to understand the phenomena and suggest solutions for the problems. The level of information in medical registers will determine the usefulness of the information that can be offered and will thus guarantee patient safety while solving the problem of detecting important events [60].

## 4. The Future of Telemedicine

A telecare system consists of equipment (basically sensors and monitors) that transfers data to a communication controller, which then forwards the data to a monitoring center where they are stored in a database for subsequent access by the user. Nowadays, some of these sensors are wireless [61], and these systems can also operate from the patient’s home to provide chronic medical care.

### 4.1. Telemedicine in Emergencies

Telemedicine is normally used to compensate for the limitations of medical resources, in which a remotely located physician provides care for a patient either in-hospital or elsewhere. However, this is not the only scenario in which telemedicine can be used. In cities, patients can usually reach emergency services within half an hour. In rural areas, however, the distances are greater and the provision of medical attention may be delayed [62]. The impact of delays in attending trauma victims has been studied for nearly a century. An analysis of the mortality of French soldiers in World War I found that 10% of those resuscitated within an hour of being wounded died, compared to 75% of those resuscitated within 8 hours [63]. These data seem to have influenced Adams Cowley in his definition of the “golden hour” concept. Even though there is no hard evidence for a particular time frame [64], it is clear that delays in resuscitation have a significant impact on patient outcome. Rapìd attention is critical not just for trauma victims but for cardiovascular, neurological and respiratory emergencies, and also for septic diseases.

In this latter scenario, telemedicine can help to shorten the time needed to provide the best standard care to the patient. In a population-based retrospective cohort study of all trauma deaths in the province of Ontario (Canada) between 2002 and 2003, Gomez *et al*. [65] found that more than half of the deaths occurred before patients reached the emergency department (ED): out-hospital deaths were twice as likely in rural locations and in areas with limited access to timely trauma center care. Moreover, among patients surviving long enough to reach hospital, there was a threefold increase in the risk of Emergency Department death in those injured in a region with limited access to trauma center care.

At present, on reaching the scene of the emergency, paramedics must make a voice call to the coordination center and transmit information to the on-call physician if they need medical advice before taking a decision. Inevitably this “second hand” information is limited and partial, and there is a strong risk that potentially valuable information will be lost. In a telemedicine service model, the physician at the coordination center has access to signals and measurements from the patient monitor and the mechanical ventilator via the video and two-way audio systems of the ambulance cabin. So, the decision-making process is based on objective information (as in the hands-on paradigm). Furthermore, if the patient needs to be hospitalized, control can be transferred to another physician located at the reference hospital who can start managing the case when patient is still on the road.

Medical emergencies are a growing problem. Statistics from the Department of Health of the United Kingdom show that emergency consultations have increased at an annual rate of 6% since 1994. In fact, excluding admissions for births, 55% of all hospital admissions go through emergency departments. [66] It is well known that delays in diagnosis and treatment are life-threatening situations. The benefits of reducing the interval from the injury until the best standard care is instituted are obvious, and this window period can clearly be reduced by the use of telemedicine systems.

### 4.2. Telemedicine as an Alternative for Treating Chronic Outpatients

Around 15% of nursing visits can be replaced by home telenursing [67]. This alternative is particularly helpful in rural patients [68]. The most relevant aspects of these technical solutions are the simplicity of operation and management, availability, reliability, and affordability [69]. Specifically in chronic respiratory patients, one relevant parameter to evaluate is the extent of the airway obstruction and its consequences for patients’ oxygenation. Regular testing is often useful in this regard. In Bratton’s study [70], 82% of physicians found telecare technology useful as a part of primary-care services, but only 45% felt that it could adequately offer a reliable assessment. On the other hand, 61% of the patients felt comfortable using the system and 94% did not believe that the technology had negative effects on their relationship with their healthcare providers [71]. Other studies have confirmed the high degree of satisfaction with this approach among respiratory patients. In an Italian study [72,73], a telemonitoring program decreased the number of hospital admissions by around 50%. These results were obtained with relatively simple equipment, and measurements were performed only twice a week. Although home care programs controlled by telemonitoring are relatively rare at present, promising results have been reported in programs with simple internet-based approaches [74], and their progressive implementation in the future seems likely. In fact, in Spain, Corral *et al*. [75,76] have proposed a model for specific application in pulmonary care, encompassing not only medical or nursing teleconsultation but also forced spirometry, the monitorization of respiratory parameters and respiratory rehabilitation.

## Figures and Tables

**Table 1.** Drawbacks of traditional devices for transcutaneous measurement of carbon dioxide.

Burns resulting from the high temperature of the electrodes
Skin abrasion resulting from excessive friction of the electrodes
Unreliable readings in patients with acidosis
Long times for calibration and stabilization
Need to change application site of the electrodes every 2–4 hours

**Table 2.** Abnormalities in the capnogram.

	**Abnormality**	**Setting**
***Inspiratory phase (0)***	CO_2_ > 0%	Rebreathing
***Phase I***	Increased length of phase I	Increased dead space Excessive PEEP
***Phase II***	Decreased phase II slope	Decreased perfusion (PTE, excessive PEEP, low cardiac output)
***Phase III***	Increased phase III slope	Inhomogeneous V/Q: - Airway obstruction - Suboptimal PEEP
***α angle***	Increased angle	The same situations that increase the slope of phase III
***β angle***	Increased angle	Reinhalation of alveolar gas: malfunctioning expiratory valve

## Abbreviations

PaO_2_: arterial oxygen partial pressure
PaCO_2_: arterial anhydride carbonic acid pressure
ABG: arterial blood gas
SpO_2_: transcutaneous arterial oxygen saturation
SaO_2_: arterial oxygen saturation obtained from arterial blood gas analysis
TcCO_2_: transcutaneous partial pressure of carbon dioxide
FEV_1_: forced spirometric value in the first second
FVC: forced vital capacity
PFTs: pulmonary function testings
*V̇*: flow over time
V: volume
Prs: pressure applied to the respiratory system
Pmus: pressure developed by the respiratory muscles
Pao: pressure at the airway opening
Pres: frictional forces generated with gas flow
Rrs: resistance of the respiratory system
Pel: elastic forces
Crs: respiratory system compliance
PEEP: positive end-expiratory pressure
PEEPi.: Intrinsic PEEP
Pmax: highest peak at end-inspiration
Pst, rs: static pressure of the respiratory system
Palv: alveolar pressure
NIV: non-invasive ventilation
ALI/ARDS: acute lung injury/adult respiratory distress syndrome
